# Bis­(4-benzoyl-3-methyl-1-phenyl-4,5-di­hydro-1*H*-pyrazol-5-olato-κ^2^ *O*,*O*′)(methanol-κ*O*)dioxidouranium(VI) methanol monosolvate

**DOI:** 10.1107/S1600536812011075

**Published:** 2012-03-21

**Authors:** Ouarda Dehbi, Saida Keraghel, Sofiane Bouacida, Fatiha Benghanem, Ali Ourari

**Affiliations:** a Laboratoire d’Electrochimie, d’Ingénierie Moléculaire et de Catalyse Redox (LEIMCR), Faculté des Sciences de l’Ingénieur, Université Farhat Abbas, Sétif 19000, Algeria; b Unité de Recherche de Chimie de l’Environnement et Moléculaire Structurale, CHEMS, Université Mentouri-Constantine, 25000 Algeria

## Abstract

In the title compound, [U(C_17_H_13_N_2_O_2_)_2_O_2_(CH_3_OH)]·CH_3_OH, the U^VI^ ion is coordinated by seven O atoms in a distorted pentagonal–bipyramidal geometry with two 3-methyl-1-phenyl-4-benzoyl-4,5-dihydro-1*H*-pyrazol-5-olate groups with two O atoms in a bidentate chelating coordination mode and by three O atoms, one of which is from a methanol ligand. The crystal packing can be described by alternating layers of complex mol­ecules along the *a* axis. The structure is stabilized by O—H⋯N and O—H⋯O hydrogen bonding and van der Waals inter­actions.

## Related literature

For the synthesis and applications of similar compounds: Okafor (1981 ▶); Caruso *et al.* (2000 ▶); Li *et al.* (1997 ▶); Zhou *et al.* (1999 ▶).
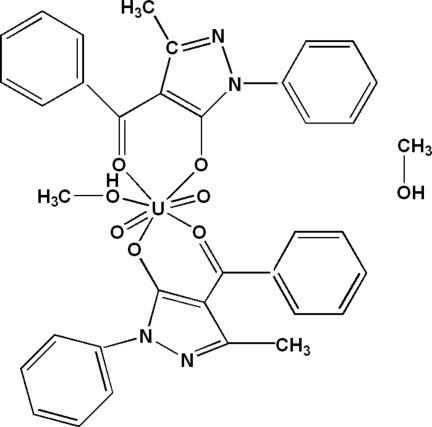


## Experimental

### 

#### Crystal data

[U(C_17_H_13_N_2_O_2_)_2_O_2_(CH_4_O)]·CH_4_O
*M* *_r_* = 888.7
Triclinic, 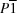

*a* = 10.3353 (18) Å
*b* = 12.988 (2) Å
*c* = 13.955 (3) Å
α = 69.938 (3)°
β = 81.728 (2)°
γ = 70.722 (3)°
*V* = 1659.9 (5) Å^3^
*Z* = 2
Mo *K*α radiation
μ = 4.95 mm^−1^
*T* = 173 K
0.40 × 0.40 × 0.30 mm

#### Data collection

Bruker APEXII diffractometer
Absorption correction: multi-scan (*SADABS*; Sheldrick, 2002) *T*_min_ = 0.242, *T*_max_ = 0.318
20251 measured reflections
10125 independent reflections
8127 reflections with *I* > 2σ(*I*)
*R*_int_ = 0.064

#### Refinement

*R*[*F* ^2^ > 2σ(*F* ^2^)] = 0.045
*wR*(*F* ^2^) = 0.096
*S* = 1.00
10125 reflections
450 parameters
1 restraint
H atoms treated by a mixture of independent and constrained refinement
Δρ_max_ = 2.45 e Å^−3^
Δρ_min_ = −3.52 e Å^−3^

### 

Data collection: *APEX2* (Bruker, 2006 ▶); cell refinement: *SAINT* (Bruker, 2006 ▶); data reduction: *SAINT*; program(s) used to solve structure: *SIR2002* (Burla *et al.*, 2005 ▶); program(s) used to refine structure: *SHELXL97* (Sheldrick, 2008 ▶); molecular graphics: *ORTEP-3 for Windows* (Farrugia, 1997 ▶) and *DIAMOND* (Brandenburg & Berndt, 2001 ▶); software used to prepare material for publication: *WinGX* (Farrugia, 1999 ▶).

## Supplementary Material

Crystal structure: contains datablock(s) global, I. DOI: 10.1107/S1600536812011075/bq2342sup1.cif

Structure factors: contains datablock(s) I. DOI: 10.1107/S1600536812011075/bq2342Isup2.hkl

Additional supplementary materials:  crystallographic information; 3D view; checkCIF report

## Figures and Tables

**Table 1:** Selected bond lengths (Å)

O1—U1	2.334 (3)
O2—U1	2.388 (3)
O3—U1	2.391 (3)
O4—U1	2.388 (3)
O5—U1	1.768 (3)
O6—U1	1.761 (3)
O7—U1	2.456 (3)

**Table 2:** Hydrogen-bond geometry (Å, °)

*D*—H⋯*A*	*D*—H	H⋯*A*	*D*⋯*A*	*D*—H⋯*A*
O7—H7*O*⋯O36*S*^i^	0.81 (2)	1.84 (5)	2.633 (6)	168 (5)
O36*S*—H36*S*⋯N2^ii^	0.82	2.12	2.878 (6)	153
C6—H6⋯O3	0.93	2.33	2.909 (7)	120
C19—H19⋯O1	0.93	2.54	2.989 (7)	110

Symmetry codes: (i) 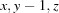; (ii) 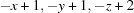.

**Fig. 1.**
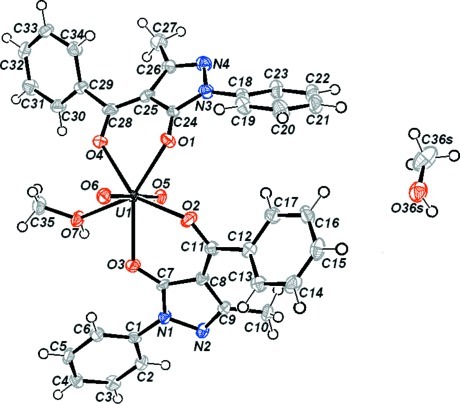
The asymmetric unit of (I) with the atom-labeling scheme. Displacement ellipsoids are drawn at the 50% probability level.

**Fig. 2.**
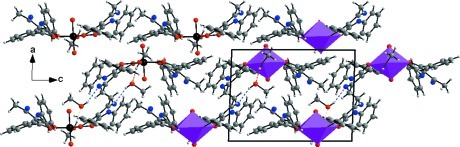
Altering layers of (I) viewed via b axis showing hydrogen bond interactions(O—H···N), in blue, as dashed lines.

## supplementary crystallographic information

### Comment

The ligands derived fromβ-diketone compounds such as those of 3-methyl-1-phenyl-4-benzoylpyrazol-5-one (H1MPBP) have been found to have good extractive ability for heavy metals traces and interesting biological activities (Okafor *et al.*, 1981). Their metal complexes M^II^–(MPBP)_2_ of copper(II) and zinc(II) are well known in the literature for their diverse therapeutic applications as anticarcinogenic, anti-inflammatory and analgesic activities (Caruso *et al.*, 2000; Li *et al.*, 1997; Zhou *et al.*, 1999). Thus, we report here the synthesis of title compound and its crystal structure. The asymetric unit of structure of (I), and the atomic numbering used, is illustrated in Fig. 1.

The U^VI^ ion is coordinated in a irregular octahedral geometry by two (3-Methyl-1-phenyl-4-benzoylpyrazol-5-one)groups with two O atoms in bidentate chelating coordination and three O atoms, when one O atom is linked to methanol moiety. One molecule of methanol is cocrystalized with the title complexe. The bond lengths for co-ordination U(V) sphere is ranging from 1.760 (3) to 2.391 (3)Å for Cu-O distances (Table 2). The crystal packing in the title structure can be described by alterning layers of complexe along the a axis (Fig. 2). It's stabilized by intermolecular O-H···N, O-H···O hydrogen bonding (Table 1, Fig. 2) and a Van Der Waals interactions. These interactions link the molecules within the layers and also link the layers together and reinforcing the cohesion of the structure.

### Experimental

106 mg (0.25 mmol) of dioxouranyl(II) acetate dihydrate (UO_2_(OAc)_2_,2H_2_O) were dissolved in 15 ml of methanol. This solution was drop wisely added, under stirring, to a methanolic solution (10 ml) containing 139 mg of 3-methyl-1-phenyl-4-benzoylpyrazol-5-one (0.5 mmol, H1MPBP). This mixture was refluxed during one night after which is abandoned for several weeks until the formation of suitable crystals. These crystals, recovered by filtration, were then washed several times with methanol and dried to yield 132 mg (60%) of the title compound.

### Refinement

The remaining H atoms were localized on Fourier maps but introduced in calculated positions and treated as riding on their parent atoms (C and O) with C—H = 0.96 Å (methyl) or 0.93 Å (aromatic) and O—H = 0.82 Å with U_iso_(H) = 1.2U_eq_(C_aromatic_) or U_iso_(H) = 1.5U_eq_(C_methyl_ and O_hydroxy_). In exept the H7o atom was located in difference Fourier maps and their coordinates were refined; the O-H7o distance was restrained to 0.82 (2)Å. The maxima and minima in the residual electron density are associated with atom U1.

### Crystal data

**Table:**

[U(C_17_H_13_N_2_O_2_)_2_O_2_(CH_4_O)]·CH_4_O	*Z* = 2
*M**_r_* = 888.7	*F*(000) = 868
Triclinic, *P*1	*D*_x_ = 1.778 Mg m^−^^3^
Hall symbol: -P 1	Mo *K*α radiation, λ = 0.71073 Å
*a* = 10.3353 (18) Å	Cell parameters from 5297 reflections
*b* = 12.988 (2) Å	θ = 2.4–26.8°
*c* = 13.955 (3) Å	µ = 4.95 mm^−^^1^
α = 69.938 (3)°	*T* = 173 K
β = 81.728 (2)°	Block, orange
γ = 70.722 (3)°	0.40 × 0.40 × 0.30 mm
*V* = 1659.9 (5) Å^3^	

### Data collection

**Table:**

Bruker APEXII diffractometer	10125 independent reflections
Radiation source: fine-focus sealed tube	8127 reflections with *I* > 2σ(*I*)
Graphite monochromator	*R*_int_ = 0.064
phi and ω scans	θ_max_ = 30.8°, θ_min_ = 2.3°
Absorption correction: multi-scan (*SADABS*; Sheldrick, 2002)	*h* = −14→14
*T*_min_ = 0.242, *T*_max_ = 0.318	*k* = −18→18
20251 measured reflections	*l* = −20→19

### Refinement

**Table:**

Refinement on *F*^2^	Primary atom site location: structure-invariant direct methods
Least-squares matrix: full	Secondary atom site location: difference Fourier map
*R*[*F*^2^ > 2σ(*F*^2^)] = 0.045	Hydrogen site location: inferred from neighbouring sites
*wR*(*F*^2^) = 0.096	H atoms treated by a mixture of independent and constrained refinement
*S* = 1.00	*w* = 1/[σ^2^(*F*_o_^2^) + (0.0322*P*)^2^] where *P* = (*F*_o_^2^ + 2*F*_c_^2^)/3
10125 reflections	(Δ/σ)_max_ = 0.002
450 parameters	Δρ_max_ = 2.45 e Å^−^^3^
1 restraint	Δρ_min_ = −3.52 e Å^−^^3^

### Special details

**Table:**

Geometry. All esds (except the esd in the dihedral angle between two l.s. planes) are estimated using the full covariance matrix. The cell esds are taken into account individually in the estimation of esds in distances, angles and torsion angles; correlations between esds in cell parameters are only used when they are defined by crystal symmetry. An approximate (isotropic) treatment of cell esds is used for estimating esds involving l.s. planes.
Refinement. Refinement of F^2^ against ALL reflections. The weighted R-factor wR and goodness of fit S are based on F^2^, conventional R-factors R are based on F, with F set to zero for negative F^2^. The threshold expression of F^2^ > 2sigma(F^2^) is used only for calculating R-factors(gt) etc. and is not relevant to the choice of reflections for refinement. R-factors based on F^2^ are statistically about twice as large as those based on F, and R- factors based on ALL data will be even larger.

### Fractional atomic coordinates and isotropic or equivalent isotropic displacement parameters (Å^2^)

**Table:**

	*x*	*y*	*z*	*U*_iso_*/*U*_eq_	
C1	0.3016 (5)	0.0112 (4)	1.0673 (3)	0.0181 (9)	
C2	0.4041 (5)	−0.0758 (5)	1.1291 (4)	0.0269 (11)	
H2	0.4829	−0.0614	1.1386	0.032*	
C3	0.3878 (5)	−0.1836 (4)	1.1761 (4)	0.0247 (11)	
H3	0.4567	−0.2416	1.2166	0.03*	
C4	0.2714 (5)	−0.2063 (4)	1.1640 (4)	0.0252 (11)	
H4	0.2608	−0.2787	1.1971	0.03*	
C5	0.1707 (5)	−0.1207 (4)	1.1023 (4)	0.0254 (11)	
H5	0.0926	−0.1363	1.093	0.03*	
C6	0.1834 (5)	−0.0119 (4)	1.0541 (4)	0.0232 (10)	
H6	0.1142	0.0453	1.0133	0.028*	
C7	0.2455 (5)	0.2101 (4)	0.9434 (3)	0.0177 (9)	
C8	0.2818 (5)	0.3085 (4)	0.9380 (4)	0.0194 (10)	
C9	0.3811 (5)	0.2681 (4)	1.0143 (4)	0.0207 (10)	
C10	0.4678 (6)	0.3295 (5)	1.0350 (4)	0.0297 (12)	
H10A	0.5279	0.2783	1.0894	0.045*	
H10B	0.41	0.3946	1.0542	0.045*	
H10C	0.5213	0.355	0.9746	0.045*	
C11	0.2170 (5)	0.4193 (4)	0.8721 (4)	0.0199 (10)	
C12	0.2119 (5)	0.5265 (4)	0.8918 (4)	0.0210 (10)	
C13	0.1680 (6)	0.5421 (5)	0.9852 (4)	0.0283 (12)	
H13	0.1491	0.4821	1.039	0.034*	
C14	0.1516 (6)	0.6457 (5)	1.0000 (4)	0.0316 (12)	
H14	0.1197	0.6564	1.0627	0.038*	
C15	0.1831 (5)	0.7329 (5)	0.9206 (5)	0.0319 (13)	
H15	0.1741	0.8023	0.9304	0.038*	
C16	0.2276 (5)	0.7186 (4)	0.8270 (4)	0.0289 (12)	
H16	0.2487	0.7781	0.7741	0.035*	
C17	0.2413 (5)	0.6159 (4)	0.8112 (4)	0.0258 (11)	
H17	0.2697	0.6067	0.7475	0.031*	
C18	0.2910 (5)	0.6501 (4)	0.4807 (4)	0.0220 (10)	
C19	0.1797 (5)	0.7099 (4)	0.5284 (4)	0.0277 (11)	
H19	0.0975	0.692	0.5404	0.033*	
C20	0.1938 (6)	0.7969 (5)	0.5578 (5)	0.0343 (13)	
H20	0.1209	0.8373	0.5911	0.041*	
C21	0.3159 (6)	0.8245 (5)	0.5380 (5)	0.0343 (13)	
H21	0.3244	0.883	0.5581	0.041*	
C22	0.4226 (5)	0.7662 (5)	0.4893 (4)	0.0300 (12)	
H22	0.5036	0.7858	0.4757	0.036*	
C23	0.4125 (5)	0.6786 (5)	0.4600 (4)	0.0299 (12)	
H23	0.4862	0.6388	0.4268	0.036*	
C24	0.2139 (5)	0.4804 (4)	0.4970 (4)	0.0207 (10)	
C25	0.2349 (5)	0.4098 (4)	0.4349 (4)	0.0216 (10)	
C26	0.3228 (5)	0.4549 (4)	0.3511 (4)	0.0232 (10)	
C27	0.3865 (6)	0.4137 (5)	0.2632 (4)	0.0369 (14)	
H27A	0.4586	0.447	0.2322	0.055*	
H27B	0.3182	0.436	0.2139	0.055*	
H27C	0.4237	0.3314	0.2867	0.055*	
C28	0.1815 (5)	0.3184 (4)	0.4603 (4)	0.0233 (10)	
C29	0.1708 (5)	0.2638 (4)	0.3842 (4)	0.0224 (10)	
C30	0.2010 (5)	0.1454 (4)	0.4139 (4)	0.0274 (11)	
H30	0.2326	0.101	0.479	0.033*	
C31	0.1844 (6)	0.0924 (5)	0.3468 (4)	0.0320 (12)	
H31	0.2052	0.0128	0.3669	0.038*	
C32	0.1375 (5)	0.1580 (5)	0.2513 (4)	0.0276 (11)	
H32	0.1279	0.1228	0.2061	0.033*	
C33	0.1043 (5)	0.2758 (5)	0.2219 (4)	0.0276 (11)	
H33	0.0704	0.3197	0.1574	0.033*	
C34	0.1209 (5)	0.3296 (4)	0.2878 (4)	0.0261 (11)	
H34	0.0988	0.4093	0.2674	0.031*	
C35	0.0585 (6)	0.0644 (5)	0.7008 (4)	0.0328 (13)	
H35A	−0.0238	0.1242	0.6743	0.049*	
H35B	0.035	0.0017	0.7528	0.049*	
H35C	0.1107	0.0377	0.6467	0.049*	
C36S	0.4590 (7)	0.9616 (7)	0.7067 (5)	0.0525 (18)	
H36A	0.5134	1.0098	0.7042	0.079*	
H36B	0.4022	0.9956	0.6484	0.079*	
H36C	0.5183	0.8871	0.7063	0.079*	
N1	0.3146 (4)	0.1229 (3)	1.0221 (3)	0.0199 (8)	
N2	0.4026 (4)	0.1577 (3)	1.0642 (3)	0.0206 (8)	
N3	0.2801 (4)	0.5596 (4)	0.4498 (3)	0.0246 (9)	
N4	0.3513 (4)	0.5416 (4)	0.3602 (3)	0.0263 (9)	
O1	0.1436 (3)	0.4773 (3)	0.5828 (3)	0.0221 (7)	
O2	0.1534 (4)	0.4329 (3)	0.7957 (3)	0.0257 (8)	
O3	0.1624 (3)	0.2027 (3)	0.8890 (2)	0.0225 (7)	
O4	0.1342 (3)	0.2794 (3)	0.5502 (3)	0.0242 (8)	
O5	0.3227 (3)	0.2567 (3)	0.6995 (3)	0.0232 (7)	
O6	−0.0384 (3)	0.3501 (3)	0.7243 (3)	0.0223 (7)	
O7	0.1379 (4)	0.1080 (3)	0.7434 (3)	0.0253 (8)	
H7O	0.207 (4)	0.058 (4)	0.768 (4)	0.038*	
O36S	0.3752 (4)	0.9501 (3)	0.7970 (3)	0.0358 (9)	
H36S	0.4216	0.9349	0.8458	0.054*	
U1	0.141702 (17)	0.305351 (14)	0.710418 (13)	0.01446 (5)	

### Atomic displacement parameters (Å^2^)

**Table:**

	*U*^11^	*U*^22^	*U*^33^	*U*^12^	*U*^13^	*U*^23^
C1	0.021 (2)	0.019 (2)	0.014 (2)	−0.0062 (18)	0.0028 (18)	−0.0060 (18)
C2	0.024 (2)	0.032 (3)	0.028 (3)	−0.011 (2)	−0.006 (2)	−0.008 (2)
C3	0.018 (2)	0.019 (2)	0.029 (3)	0.0030 (19)	−0.006 (2)	−0.004 (2)
C4	0.029 (3)	0.018 (2)	0.028 (3)	−0.009 (2)	0.001 (2)	−0.006 (2)
C5	0.028 (3)	0.027 (3)	0.028 (3)	−0.012 (2)	−0.007 (2)	−0.011 (2)
C6	0.021 (2)	0.024 (2)	0.023 (3)	−0.006 (2)	−0.0052 (19)	−0.004 (2)
C7	0.021 (2)	0.023 (2)	0.011 (2)	−0.0065 (19)	−0.0016 (17)	−0.0072 (19)
C8	0.020 (2)	0.018 (2)	0.020 (2)	−0.0045 (18)	−0.0037 (18)	−0.0059 (19)
C9	0.022 (2)	0.022 (2)	0.019 (2)	−0.0074 (19)	−0.0028 (19)	−0.007 (2)
C10	0.037 (3)	0.028 (3)	0.032 (3)	−0.017 (2)	−0.011 (2)	−0.007 (2)
C11	0.022 (2)	0.018 (2)	0.021 (2)	−0.0060 (19)	−0.0009 (19)	−0.0061 (19)
C12	0.019 (2)	0.021 (2)	0.023 (3)	−0.0024 (19)	−0.0031 (19)	−0.010 (2)
C13	0.037 (3)	0.025 (3)	0.026 (3)	−0.014 (2)	−0.001 (2)	−0.007 (2)
C14	0.038 (3)	0.038 (3)	0.027 (3)	−0.013 (3)	0.003 (2)	−0.021 (3)
C15	0.031 (3)	0.025 (3)	0.047 (4)	−0.007 (2)	−0.007 (3)	−0.020 (3)
C16	0.032 (3)	0.021 (2)	0.035 (3)	−0.013 (2)	−0.003 (2)	−0.005 (2)
C17	0.027 (3)	0.022 (2)	0.027 (3)	−0.007 (2)	−0.004 (2)	−0.006 (2)
C18	0.020 (2)	0.016 (2)	0.027 (3)	−0.0031 (18)	−0.0045 (19)	−0.002 (2)
C19	0.027 (3)	0.025 (3)	0.032 (3)	−0.012 (2)	0.003 (2)	−0.007 (2)
C20	0.033 (3)	0.026 (3)	0.045 (4)	−0.009 (2)	0.007 (3)	−0.015 (3)
C21	0.043 (3)	0.020 (3)	0.042 (4)	−0.013 (2)	−0.013 (3)	−0.005 (2)
C22	0.022 (2)	0.029 (3)	0.039 (3)	−0.011 (2)	−0.005 (2)	−0.007 (2)
C23	0.023 (2)	0.027 (3)	0.041 (3)	−0.010 (2)	−0.002 (2)	−0.010 (2)
C24	0.022 (2)	0.014 (2)	0.021 (2)	−0.0067 (18)	−0.0126 (19)	0.0060 (19)
C25	0.017 (2)	0.026 (2)	0.019 (2)	−0.0004 (19)	−0.0069 (18)	−0.007 (2)
C26	0.018 (2)	0.027 (3)	0.022 (3)	−0.007 (2)	−0.0014 (19)	−0.004 (2)
C27	0.037 (3)	0.048 (4)	0.031 (3)	−0.019 (3)	0.006 (2)	−0.015 (3)
C28	0.022 (2)	0.020 (2)	0.020 (3)	−0.0007 (19)	−0.0089 (19)	0.001 (2)
C29	0.023 (2)	0.024 (2)	0.020 (3)	−0.007 (2)	−0.0035 (19)	−0.006 (2)
C30	0.035 (3)	0.023 (3)	0.023 (3)	−0.007 (2)	−0.005 (2)	−0.006 (2)
C31	0.039 (3)	0.023 (3)	0.035 (3)	−0.009 (2)	0.000 (2)	−0.011 (2)
C32	0.023 (2)	0.040 (3)	0.030 (3)	−0.014 (2)	0.004 (2)	−0.020 (3)
C33	0.024 (2)	0.041 (3)	0.019 (3)	−0.013 (2)	−0.004 (2)	−0.007 (2)
C34	0.023 (2)	0.023 (2)	0.029 (3)	−0.006 (2)	−0.003 (2)	−0.003 (2)
C35	0.037 (3)	0.035 (3)	0.036 (3)	−0.019 (3)	−0.002 (2)	−0.015 (3)
C36S	0.039 (4)	0.065 (5)	0.058 (5)	0.001 (3)	−0.015 (3)	−0.036 (4)
N1	0.0207 (19)	0.0183 (19)	0.021 (2)	−0.0052 (16)	−0.0050 (16)	−0.0057 (17)
N2	0.0175 (18)	0.022 (2)	0.023 (2)	−0.0059 (16)	−0.0029 (16)	−0.0081 (18)
N3	0.022 (2)	0.028 (2)	0.023 (2)	−0.0094 (18)	0.0007 (17)	−0.0063 (19)
N4	0.021 (2)	0.030 (2)	0.026 (2)	−0.0088 (18)	0.0007 (17)	−0.0058 (19)
O1	0.0249 (17)	0.0203 (17)	0.0211 (18)	−0.0100 (14)	0.0050 (14)	−0.0061 (14)
O2	0.035 (2)	0.0194 (17)	0.0234 (19)	−0.0055 (15)	−0.0116 (15)	−0.0062 (15)
O3	0.0279 (18)	0.0166 (16)	0.0206 (18)	−0.0054 (14)	−0.0072 (14)	−0.0019 (14)
O4	0.0287 (18)	0.0280 (19)	0.0178 (18)	−0.0139 (16)	−0.0002 (14)	−0.0047 (15)
O5	0.0158 (15)	0.0296 (19)	0.0270 (19)	−0.0092 (14)	−0.0029 (14)	−0.0091 (16)
O6	0.0168 (15)	0.0244 (18)	0.0248 (19)	−0.0058 (14)	−0.0041 (13)	−0.0057 (15)
O7	0.0279 (19)	0.0160 (17)	0.032 (2)	−0.0026 (14)	−0.0136 (16)	−0.0064 (16)
O36S	0.032 (2)	0.030 (2)	0.045 (3)	−0.0058 (17)	−0.0157 (18)	−0.009 (2)
U1	0.01361 (8)	0.01473 (8)	0.01518 (9)	−0.00454 (6)	−0.00239 (6)	−0.00391 (6)

### Geometric parameters (Å, º)

**Table:**

C1—C2	1.395 (7)	C22—H22	0.93
C1—C6	1.400 (6)	C23—H23	0.93
C1—N1	1.413 (6)	C24—O1	1.304 (6)
C2—C3	1.383 (7)	C24—N3	1.353 (6)
C2—H2	0.93	C24—C25	1.413 (7)
C3—C4	1.375 (7)	C25—C28	1.388 (7)
C3—H3	0.93	C25—C26	1.454 (7)
C4—C5	1.378 (7)	C26—N4	1.302 (7)
C4—H4	0.93	C26—C27	1.486 (7)
C5—C6	1.383 (7)	C27—H27A	0.96
C5—H5	0.93	C27—H27B	0.96
C6—H6	0.93	C27—H27C	0.96
C7—O3	1.271 (5)	C28—O4	1.273 (6)
C7—N1	1.355 (6)	C28—C29	1.498 (7)
C7—C8	1.422 (6)	C29—C30	1.384 (7)
C8—C11	1.414 (6)	C29—C34	1.388 (7)
C8—C9	1.427 (6)	C30—C31	1.396 (8)
C9—N2	1.319 (6)	C30—H30	0.93
C9—C10	1.492 (7)	C31—C32	1.368 (7)
C10—H10A	0.96	C31—H31	0.93
C10—H10B	0.96	C32—C33	1.374 (8)
C10—H10C	0.96	C32—H32	0.93
C11—O2	1.262 (6)	C33—C34	1.389 (7)
C11—C12	1.493 (7)	C33—H33	0.93
C12—C13	1.377 (7)	C34—H34	0.93
C12—C17	1.394 (7)	C35—O7	1.424 (6)
C13—C14	1.383 (7)	C35—H35A	0.96
C13—H13	0.93	C35—H35B	0.96
C14—C15	1.376 (8)	C35—H35C	0.96
C14—H14	0.93	C36S—O36S	1.415 (8)
C15—C16	1.375 (8)	C36S—H36A	0.96
C15—H15	0.93	C36S—H36B	0.96
C16—C17	1.383 (7)	C36S—H36C	0.96
C16—H16	0.93	N1—N2	1.409 (5)
C17—H17	0.93	N3—N4	1.408 (6)
C18—C19	1.383 (7)	O1—U1	2.334 (3)
C18—C23	1.389 (7)	O2—U1	2.388 (3)
C18—N3	1.426 (6)	O3—U1	2.391 (3)
C19—C20	1.382 (8)	O4—U1	2.388 (3)
C19—H19	0.93	O5—U1	1.768 (3)
C20—C21	1.388 (7)	O6—U1	1.761 (3)
C20—H20	0.93	O7—U1	2.456 (3)
C21—C22	1.355 (8)	O7—H7O	0.811 (19)
C21—H21	0.93	O36S—H36S	0.82
C22—C23	1.371 (7)		
			
C2—C1—C6	119.4 (4)	N4—C26—C27	118.0 (4)
C2—C1—N1	120.1 (4)	C25—C26—C27	129.8 (5)
C6—C1—N1	120.4 (4)	C26—C27—H27A	109.5
C3—C2—C1	119.6 (5)	C26—C27—H27B	109.5
C3—C2—H2	120.2	H27A—C27—H27B	109.5
C1—C2—H2	120.2	C26—C27—H27C	109.5
C4—C3—C2	121.1 (5)	H27A—C27—H27C	109.5
C4—C3—H3	119.4	H27B—C27—H27C	109.5
C2—C3—H3	119.4	O4—C28—C25	120.7 (5)
C3—C4—C5	119.2 (5)	O4—C28—C29	116.6 (4)
C3—C4—H4	120.4	C25—C28—C29	122.7 (4)
C5—C4—H4	120.4	C30—C29—C34	119.4 (5)
C4—C5—C6	121.2 (5)	C30—C29—C28	118.9 (4)
C4—C5—H5	119.4	C34—C29—C28	121.5 (5)
C6—C5—H5	119.4	C29—C30—C31	120.3 (5)
C5—C6—C1	119.4 (4)	C29—C30—H30	119.8
C5—C6—H6	120.3	C31—C30—H30	119.8
C1—C6—H6	120.3	C32—C31—C30	119.7 (5)
O3—C7—N1	125.3 (4)	C32—C31—H31	120.2
O3—C7—C8	128.4 (4)	C30—C31—H31	120.2
N1—C7—C8	106.3 (4)	C31—C32—C33	120.4 (5)
C11—C8—C7	122.2 (4)	C31—C32—H32	119.8
C11—C8—C9	132.6 (4)	C33—C32—H32	119.8
C7—C8—C9	105.1 (4)	C32—C33—C34	120.5 (5)
N2—C9—C8	111.4 (4)	C32—C33—H33	119.7
N2—C9—C10	119.2 (4)	C34—C33—H33	119.7
C8—C9—C10	129.1 (4)	C29—C34—C33	119.6 (5)
C9—C10—H10A	109.5	C29—C34—H34	120.2
C9—C10—H10B	109.5	C33—C34—H34	120.2
H10A—C10—H10B	109.5	O7—C35—H35A	109.5
C9—C10—H10C	109.5	O7—C35—H35B	109.5
H10A—C10—H10C	109.5	H35A—C35—H35B	109.5
H10B—C10—H10C	109.5	O7—C35—H35C	109.5
O2—C11—C8	121.4 (4)	H35A—C35—H35C	109.5
O2—C11—C12	116.1 (4)	H35B—C35—H35C	109.5
C8—C11—C12	122.4 (4)	O36S—C36S—H36A	109.5
C13—C12—C17	119.7 (5)	O36S—C36S—H36B	109.5
C13—C12—C11	121.2 (4)	H36A—C36S—H36B	109.5
C17—C12—C11	118.8 (4)	O36S—C36S—H36C	109.5
C12—C13—C14	120.8 (5)	H36A—C36S—H36C	109.5
C12—C13—H13	119.6	H36B—C36S—H36C	109.5
C14—C13—H13	119.6	C7—N1—N2	111.4 (4)
C15—C14—C13	119.2 (5)	C7—N1—C1	128.8 (4)
C15—C14—H14	120.4	N2—N1—C1	119.7 (4)
C13—C14—H14	120.4	C9—N2—N1	105.8 (4)
C16—C15—C14	120.8 (5)	C24—N3—N4	111.7 (4)
C16—C15—H15	119.6	C24—N3—C18	129.4 (4)
C14—C15—H15	119.6	N4—N3—C18	118.9 (4)
C15—C16—C17	120.3 (5)	C26—N4—N3	105.4 (4)
C15—C16—H16	119.8	C24—O1—U1	122.6 (3)
C17—C16—H16	119.8	C11—O2—U1	133.4 (3)
C16—C17—C12	119.2 (5)	C7—O3—U1	122.7 (3)
C16—C17—H17	120.4	C28—O4—U1	134.8 (3)
C12—C17—H17	120.4	C35—O7—U1	131.0 (3)
C19—C18—C23	121.1 (5)	C35—O7—H7O	111 (4)
C19—C18—N3	119.7 (4)	U1—O7—H7O	115 (4)
C23—C18—N3	119.2 (5)	C36S—O36S—H36S	109.5
C20—C19—C18	118.2 (5)	O6—U1—O5	178.33 (15)
C20—C19—H19	120.9	O6—U1—O1	92.53 (13)
C18—C19—H19	120.9	O5—U1—O1	89.13 (14)
C19—C20—C21	120.6 (5)	O6—U1—O4	92.24 (14)
C19—C20—H20	119.7	O5—U1—O4	88.38 (14)
C21—C20—H20	119.7	O1—U1—O4	72.52 (12)
C22—C21—C20	120.1 (5)	O6—U1—O2	89.92 (14)
C22—C21—H21	119.9	O5—U1—O2	90.42 (14)
C20—C21—H21	119.9	O1—U1—O2	73.82 (12)
C21—C22—C23	120.8 (5)	O4—U1—O2	146.33 (12)
C21—C22—H22	119.6	O6—U1—O3	91.12 (13)
C23—C22—H22	119.6	O5—U1—O3	87.43 (14)
C22—C23—C18	119.1 (5)	O1—U1—O3	145.24 (12)
C22—C23—H23	120.4	O4—U1—O3	141.85 (11)
C18—C23—H23	120.4	O2—U1—O3	71.63 (11)
O1—C24—N3	123.2 (5)	O6—U1—O7	89.51 (14)
O1—C24—C25	129.8 (4)	O5—U1—O7	89.21 (14)
N3—C24—C25	107.1 (4)	O1—U1—O7	144.37 (12)
C28—C25—C24	122.9 (5)	O4—U1—O7	71.86 (12)
C28—C25—C26	133.4 (5)	O2—U1—O7	141.79 (12)
C24—C25—C26	103.7 (4)	O3—U1—O7	70.18 (12)
N4—C26—C25	112.1 (5)		
			
C6—C1—C2—C3	0.2 (8)	C32—C33—C34—C29	−0.3 (7)
N1—C1—C2—C3	177.5 (5)	O3—C7—N1—N2	177.7 (4)
C1—C2—C3—C4	−0.7 (8)	C8—C7—N1—N2	−4.3 (5)
C2—C3—C4—C5	1.3 (8)	O3—C7—N1—C1	−6.7 (8)
C3—C4—C5—C6	−1.2 (8)	C8—C7—N1—C1	171.4 (4)
C4—C5—C6—C1	0.7 (8)	C2—C1—N1—C7	163.4 (5)
C2—C1—C6—C5	−0.2 (7)	C6—C1—N1—C7	−19.4 (7)
N1—C1—C6—C5	−177.4 (5)	C2—C1—N1—N2	−21.3 (7)
O3—C7—C8—C11	5.4 (8)	C6—C1—N1—N2	155.9 (4)
N1—C7—C8—C11	−172.6 (4)	C8—C9—N2—N1	−0.9 (5)
O3—C7—C8—C9	−178.5 (5)	C10—C9—N2—N1	−175.0 (4)
N1—C7—C8—C9	3.5 (5)	C7—N1—N2—C9	3.3 (5)
C11—C8—C9—N2	173.9 (5)	C1—N1—N2—C9	−172.8 (4)
C7—C8—C9—N2	−1.6 (6)	O1—C24—N3—N4	178.3 (4)
C11—C8—C9—C10	−12.8 (9)	C25—C24—N3—N4	−3.0 (5)
C7—C8—C9—C10	171.7 (5)	O1—C24—N3—C18	1.5 (8)
C7—C8—C11—O2	−20.2 (7)	C25—C24—N3—C18	−179.9 (4)
C9—C8—C11—O2	164.9 (5)	C19—C18—N3—C24	−37.5 (7)
C7—C8—C11—C12	155.9 (5)	C23—C18—N3—C24	144.1 (5)
C9—C8—C11—C12	−19.0 (8)	C19—C18—N3—N4	145.8 (5)
O2—C11—C12—C13	125.6 (5)	C23—C18—N3—N4	−32.6 (7)
C8—C11—C12—C13	−50.7 (7)	C25—C26—N4—N3	−1.4 (5)
O2—C11—C12—C17	−48.9 (6)	C27—C26—N4—N3	−178.8 (4)
C8—C11—C12—C17	134.8 (5)	C24—N3—N4—C26	2.8 (5)
C17—C12—C13—C14	0.5 (8)	C18—N3—N4—C26	−180.0 (4)
C11—C12—C13—C14	−174.0 (5)	N3—C24—O1—U1	−142.0 (4)
C12—C13—C14—C15	−1.6 (8)	C25—C24—O1—U1	39.7 (6)
C13—C14—C15—C16	1.3 (8)	C8—C11—O2—U1	−12.4 (7)
C14—C15—C16—C17	0.1 (8)	C12—C11—O2—U1	171.3 (3)
C15—C16—C17—C12	−1.3 (8)	N1—C7—O3—U1	−144.5 (4)
C13—C12—C17—C16	1.0 (7)	C8—C7—O3—U1	37.9 (6)
C11—C12—C17—C16	175.5 (5)	C25—C28—O4—U1	−11.9 (7)
C23—C18—C19—C20	−1.8 (8)	C29—C28—O4—U1	170.5 (3)
N3—C18—C19—C20	179.8 (5)	C24—O1—U1—O6	−131.8 (3)
C18—C19—C20—C21	1.2 (8)	C24—O1—U1—O5	48.3 (3)
C19—C20—C21—C22	0.1 (9)	C24—O1—U1—O4	−40.3 (3)
C20—C21—C22—C23	−0.8 (9)	C24—O1—U1—O2	139.0 (4)
C21—C22—C23—C18	0.1 (8)	C24—O1—U1—O3	132.6 (3)
C19—C18—C23—C22	1.2 (8)	C24—O1—U1—O7	−39.1 (4)
N3—C18—C23—C22	179.7 (5)	C28—O4—U1—O6	123.7 (4)
O1—C24—C25—C28	−1.6 (8)	C28—O4—U1—O5	−57.9 (4)
N3—C24—C25—C28	179.8 (4)	C28—O4—U1—O1	31.7 (4)
O1—C24—C25—C26	−179.4 (5)	C28—O4—U1—O2	30.5 (5)
N3—C24—C25—C26	2.0 (5)	C28—O4—U1—O3	−141.6 (4)
C28—C25—C26—N4	−177.8 (5)	C28—O4—U1—O7	−147.6 (5)
C24—C25—C26—N4	−0.3 (6)	C11—O2—U1—O6	126.2 (4)
C28—C25—C26—C27	−0.8 (9)	C11—O2—U1—O5	−52.2 (4)
C24—C25—C26—C27	176.7 (5)	C11—O2—U1—O1	−141.2 (5)
C24—C25—C28—O4	−14.2 (7)	C11—O2—U1—O4	−139.9 (4)
C26—C25—C28—O4	162.8 (5)	C11—O2—U1—O3	35.0 (4)
C24—C25—C28—C29	163.3 (4)	C11—O2—U1—O7	37.1 (5)
C26—C25—C28—C29	−19.7 (8)	C7—O3—U1—O6	−133.2 (4)
O4—C28—C29—C30	−42.5 (7)	C7—O3—U1—O5	47.7 (4)
C25—C28—C29—C30	139.9 (5)	C7—O3—U1—O1	−37.1 (4)
O4—C28—C29—C34	132.2 (5)	C7—O3—U1—O4	131.8 (3)
C25—C28—C29—C34	−45.4 (7)	C7—O3—U1—O2	−43.6 (3)
C34—C29—C30—C31	1.4 (8)	C7—O3—U1—O7	137.8 (4)
C28—C29—C30—C31	176.2 (5)	C35—O7—U1—O6	49.0 (4)
C29—C30—C31—C32	−0.3 (8)	C35—O7—U1—O5	−132.1 (4)
C30—C31—C32—C33	−1.2 (8)	C35—O7—U1—O1	−44.6 (5)
C31—C32—C33—C34	1.5 (8)	C35—O7—U1—O4	−43.5 (4)
C30—C29—C34—C33	−1.1 (7)	C35—O7—U1—O2	138.3 (4)
C28—C29—C34—C33	−175.8 (4)	C35—O7—U1—O3	140.4 (5)

### Hydrogen-bond geometry (Å, º)

**Table:**

*D*—H···*A*	*D*—H	H···*A*	*D*···*A*	*D*—H···*A*
O7—H7*O*···O36*S*^i^	0.81 (2)	1.84 (5)	2.633 (6)	168 (5)
O36*S*—H36*S*···N2^ii^	0.82	2.12	2.878 (6)	153
C6—H6···O3	0.93	2.33	2.909 (7)	120
C19—H19···O1	0.93	2.54	2.989 (7)	110

Symmetry codes: (i) *x*, *y*−1, *z*; (ii) −*x*+1, −*y*+1, −*z*+2.
